# Emerging SARS-CoV-2 variants of concern evade humoral immune responses from infection and vaccination

**DOI:** 10.1126/sciadv.abj5365

**Published:** 2021-09-03

**Authors:** Tom G. Caniels, Ilja Bontjer, Karlijn van der Straten, Meliawati Poniman, Judith A. Burger, Brent Appelman, H. A. Ayesha Lavell, Melissa Oomen, Gert-Jan Godeke, Coralie Valle, Ramona Mögling, Hugo D. G. van Willigen, Elke Wynberg, Michiel Schinkel, Lonneke A. van Vught, Denise Guerra, Jonne L. Snitselaar, Devidas N. Chaturbhuj, Isabel Cuella Martin, Amsterdam UMC COVID-19 S3/HCW study group, John P. Moore, Menno D. de Jong, Chantal Reusken, Jonne J. Sikkens, Marije K. Bomers, Godelieve J. de Bree, Marit J. van Gils, Dirk Eggink, Rogier W. Sanders

**Affiliations:** 1 Department of Medical Microbiology, Amsterdam UMC, University of Amsterdam, Amsterdam Institute for Infection and Immunity, Amsterdam, Netherlands.; 2 Department of Internal Medicine, Amsterdam UMC, University of Amsterdam, Amsterdam Institute for Infection and Immunity, Amsterdam, Netherlands.; 3 Center for Experimental and Molecular Medicine, Amsterdam UMC, University of Amsterdam, Amsterdam Institute for Infection and Immunity, Amsterdam, Netherlands.; 4 Department of Internal Medicine, Amsterdam UMC, Vrije Universiteit Amsterdam, Amsterdam Institute for Infection and Immunity, Amsterdam, Netherlands.; 5 Centre for Infectious Disease Control, National Institute for Public Health and the Environment, Bilthoven, Netherlands.; 6 Department of Infectious Diseases, Amsterdam UMC, University of Amsterdam, Amsterdam Institute for Infection and Immunity, Amsterdam, Netherlands.; 7 Public Health Service of Amsterdam, Amsterdam, Netherlands.; 8 Department of Microbiology and Immunology, Weill Medical College of Cornell University, New York, NY, USA.

## Abstract

Emerging SARS-CoV-2 variants of concern (VOCs) pose a threat to human immunity induced by natural infection and vaccination. We assessed the recognition of three VOCs (B.1.1.7, B.1.351, and P.1) in cohorts of COVID-19 convalescent patients (*n* = 69) and Pfizer-BioNTech vaccine recipients (*n* = 50). Spike binding and neutralization against all three VOCs were substantially reduced in most individuals, with the largest four- to sevenfold reduction in neutralization being observed against B.1.351. While hospitalized patients with COVID-19 and vaccinees maintained sufficient neutralizing titers against all three VOCs, 39% of nonhospitalized patients exhibited no detectable neutralization against B.1.351. Moreover, monoclonal neutralizing antibodies show sharp reductions in their binding kinetics and neutralizing potential to B.1.351 and P.1 but not to B.1.1.7. These data have implications for the degree to which pre-existing immunity can protect against subsequent infection with VOCs and informs policy makers of susceptibility to globally circulating SARS-CoV-2 VOCs.

## Teaser

Antibodies of convalescent COVID-19 patients and vaccine recipients show reduced recognition of SARS-CoV-2 variants of concern.

## INTRODUCTION

With more than 140 million confirmed infections and more than three million deaths as of April 2021, the coronavirus disease 2019 (COVID-19) pandemic shows few signs of abating (*1*). While severe acute respiratory syndrome coronavirus 2 (SARS-CoV-2), the causative agent of COVID-19, remained relatively stable genetically and antigenically during the first stage of the pandemic, higher levels of genetic variation have been observed during the second wave of the pandemic with considerable genetic changes compared to the original Wuhan Hu-1 strain of SARS-CoV-2. These genetic changes include substitutions within the functional domains of the SARS-CoV-2 spike (S) protein resulting in altered phenotypes of the virus. The World Health Organization has currently defined three variants of concern (VOCs) based on possible increase in transmissibility or change in COVID-19 epidemiology, increase in virulence or change in clinical disease presentation, or decrease in effectiveness of available diagnostics, vaccines, and therapeutics. These VOCs include B.1.1.7 (20I/N501Y.V1, first detected in the United Kingdom), B.1.351 (20H/N501Y.V2, first detected in South Africa), and B.1.1.28.P1 (P.1, 20 J/N501Y.V3, first detected in Brazil), which have since spread globally, and as of April 2021, cases with these variants have been reported in 132, 82, and 52 countries, respectively (*2*–*5*). The emergence of these variants has raised concerns as to whether immunity, be it natural immunity from prior infection or immunity from vaccination, can protect against these different variants. Moreover, it is unclear whether therapeutic monoclonal neutralizing antibodies (NAbs) isolated from convalescent donors retain therapeutic efficacy against these variants.

**Fig. 1.**
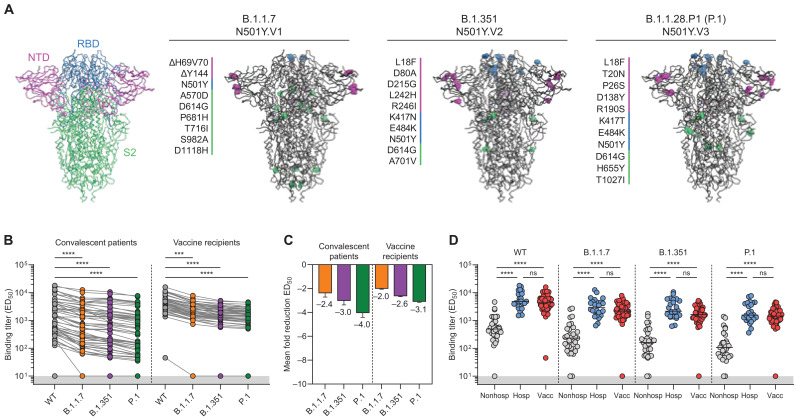
Binding of convalescent and vaccinee sera to VOCs B.1.1.7, B.1.351, and P.1 spike proteins. (**A**) Structural representation of spike (S) protein with its three domains (NTD, magenta; RBD, blue; S2 domain, green). Mutations in each of the VOC S proteins are listed and their location in the trimer. Colors correspond to the S protein domains in which the mutation occurs in. (**B**) Half-maximal binding (ED_50_) titers of polyclonal convalescent sera (left, *n* = 57) and vaccinee sera (right, *n* = 50) to S protein of B.1.1.7, B.1.351, and P.1 VOCs. Connected dots indicate results from the same individual. The lower cutoff for binding was set at an ED_50_ of 10 (gray shading). (**C**) Means ± SEM fold reductions in ED_50_ titers for convalescent patients and vaccine recipients against S proteins of B.1.1.7, B.1.351, and P.1 VOCs in comparison to ED_50_ titers to the wild-type (WT) S protein. (**D**) ED_50_ titers of nonhospitalized patients with COVID-19, hospitalized patients with COVID-19, and vaccine recipients against WT S protein and each of the VOC S protein. ****P < 0.0001 and ***P < 0.001; ns, not significant. All data points shown here represent the mean of a technical triplicate.

The S protein of coronaviruses is the main target of NAbs and consists of a membrane-proximal S2 domain containing the fusion peptide and a membrane-distal S1 domain containing the receptor binding domain (RBD) and the N-terminal domain (NTD) (Fig. 1A). The S protein mediates viral entry through interaction with the angiotensin-converting enzyme 2 (ACE2) receptor on host cells (*6*, *7*). Therefore, substitutions present in the RBD of these emerging VOCs are particularly worrisome as they might improve binding to the human receptor and thus increase viral fitness and transmissibility. B.1.1.7, B.1.351, and P.1 all share the N501Y RBD mutation, which contributes to an enhanced interaction with the human ACE2 receptor resulting in increased infectivity and transmissibility (Fig. 1A) (*8*, *9*). In addition, both B.1.351 and P.1 contain the E484K substitution and a substitution at position 417 (K417N in B.1.351 and K417T in P.1), both of which have been implicated in escape from NAbs by several studies (Fig. 1A) (*10*, *11*). More recently, E484K has also been observed in B.1.1.7 variants that have adopted this mutation independently, leading to a more substantial loss of neutralizing titers in vaccinated individuals than for B.1.1.7 alone (*12*). Additional substitutions mostly arise in the NTD region, located apically on the S1 domain. Although the exact functional implication of substitutions within the NTD is not clear, it has been shown that this domain is an important target for NAbs (*13*, *14*). VOCs B.1.351 and P.1 both carry five mutations in this region of which they share one (L18F). Early reports on the B.1.351 lineage reported variants with a substitution at position 242 and 246 (L242H and R246I), while other sublineages had a three amino acid deletion (Δ242 to 244) (*4*). B.1.1.7 does not have substitutions in its NTD but has deletions in this region (Δ69 to 70 and Δ144) (Fig. 1A). Other mutations are identified in the S2 domain of the S protein, and their impact on antibody recognition and infectivity is not yet well understood, partly because the vast majority of NAbs target the RBD or NTD. Last, all these variants have the D614G mutation that defines the B.1 lineage and became dominant throughout 2020, now being present in the large majority of sequenced SARS-CoV-2 variants (>99%) (Fig. 1A) (*15*).

All three VOCs have been linked to increased infectivity and transmissibility, and the first reports about specific substitutions in B.1.351 and P.1 associated with escape from immunity by infection or mRNA vaccines have become available (*10*, *16*–*18*). These first reports suggest that pre-existing immunity was generally sufficient to neutralize B.1.1.7 to similar levels as wild type (WT) in mRNA vaccine recipients and in convalescent individuals up to 9 months after infection (*17*, *19*). In case of B.1.351, the impact is more substantial with neutralizing titers from convalescent patients being reduced by ~6- to 13-fold, while vaccine recipients are reported to have a ~10- to 14-fold reduction (*10*, *17*, *18*). Moreover, it is reported that ~40% of convalescent patients do not have any neutralizing activity against B.1.351 9 months after primary infection (*17*). A recent study showed that although B.1.351 and P.1 have similar mutations in their RBD, sera from convalescent patients and mRNA vaccine recipients showed a neutralization reduction of ~3-fold against P.1, while this was 7- to 13-fold for B.1.351 (*20*, *21*).

Although these studies have provided valuable initial insights in altered antigenic properties of these VOCs, few studies have compared all three VOCs side by side. Furthermore, comparing immune responses from vaccine recipients and individuals who have suffered from mild or severe COVID-19 as well as reactivity of NAbs to all three VOCs within the same study will provide useful insights that can be used for additional surveillance of SARS-CoV-2 variants, the use of monoclonal antibodies for treatment, and modifications of vaccines to increase immune coverage of immunity to include yet unknown emerging SARS-CoV-2 variants.

## RESULTS

### Recognition of SARS-CoV-2 VOCs by convalescent and vaccine sera is reduced

Here, we assessed the impact of VOCs B.1.1.7, B.1.351, and P.1 on humoral immunity elicited either by mRNA vaccination or by natural infection with SARS-CoV-2. To this end, we studied two cohorts of individuals: the COVID-19 Specific Antibodies (COSCA) cohort that included convalescent patients with COVID-19 (*n* = 69) and the S3 cohort that included health care workers (HCWs) who were vaccinated twice with the Pfizer-BioNTech COVID-19 vaccine (*n* = 50) (Table 1). The COSCA cohort included hospitalized and nonhospitalized patients in the Netherlands who were enrolled and sampled 4 to 6 weeks after symptom onset (i.e., close to the expected peak of humoral immunity) between March 2020 and January 2021 (Table 1). On the basis of the rarity of B.1.351 and P.1 in the Netherlands, no individuals are expected to have been infected with either VOC. However, infections with B.1.1.7 became more prevalent toward the end of 2020, with the estimated prevalence of B.1.1.7 reaching 24% in the Netherlands in the week of the last inclusion (January 2021) (*22*). As genomic data were not collected as part of this study, we cannot exclude that a few patients were infected with B.1.1.7. The S3 cohort consists of HCWs without prior SARS-CoV-2 infection who received the approved Pfizer-BioNTech COVID-19 mRNA vaccine twice with a 3-week interval and sampled 4 weeks after the second vaccination (Table 1).

**Table 1.** Sociodemographic and clinical characteristics of study populations.

**Sociodemographic** **and clinical** **characteristics**	**Number of individuals, *n* (%)**	
	**Convalescent** **COVID-19**	**Vaccinated HCWs**
	***n* = 69**	***n* = 50**
**Sex**		
Male	33 (48)	19 (38)
Female	36 (52)	31 (62)
**Age in years**		
<35	20 (29)	14 (28)
35–60	27 (39)	32 (64)
>60	22 (32)	4 (8)
**Hospitalization**		
No	41 (59)	0 (0)
Yes		
Ward	24 (35)	
ICU	4 (6)	
**Treatment**		
Dexamethasone	12 (18)	0 (0)
Remdesivir	9 (13)	0 (0)

We first assessed S protein binding titers of convalescent and vaccinee sera in a custom multiplex protein microarray that has been extensively validated for clinical use (table S1) (*23*). We generated S proteins, using previously described stabilization approaches, from all three VOCs that had been identified at the time of assessment and a control WT S protein from the Wuhan Hu-1 virus (GenBank: MN908947.3) isolated in December 2019 (*6*, *24*, *25*). Overall, the antibody responses against each S protein were heterogeneous and differed up to ~1200-fold between the strongest and weakest responders. The recognition of the three VOCs by convalescent patients was significantly reduced compared to WT by an average of 2.4-fold, 3-fold, and 4-fold for B.1.1.7, B.1.351, and P.1, respectively (*P* < 0.0001 for all; Fig. 1, B and C). Binding titers elicited by the mRNA vaccine were more homogeneous than those elicited by natural infection and differed ~10-fold between responders, with all participants having half-maximal binding titers (ED_50_s) exceeding 10^3^, except for one poor responder (Fig. 1B). Since the variability of binding titers in convalescent sera is considerable, we examined whether this variability was related to severity of disease (i.e., hospital admission; Fig. 1D). We observed a highly significant ~8-fold difference in ED_50_s between nonhospitalized patients and hospitalized patients, which is in line with previous reports of WT SARS-CoV-2 binding titers correlating with severity of the disease (*P* < 0.0001) (*26*, *27*). This difference in binding titers between nonhospitalized patients and hospitalized patients was consistent for all three SARS-CoV-2 VOCs studied (Fig. 1D). When comparing immune responses of vaccine recipients with convalescent sera, we observed similar S protein binding titers between vaccinee and hospitalized patients, which are an average ~4- to 11-fold higher compared to nonhospitalized patients for all VOCs (Fig. 1D). Together, these data indicate that vaccine recipients and patients with COVID-19 exhibit reduced binding to S proteins of the currently circulating VOCs, with hospitalized patients and vaccine recipients exhibiting higher binding titers overall compared to nonhospitalized patients.

### SARS-CoV-2 VOCs are substantially less sensitive to serum NAbs

**Fig. 2.**
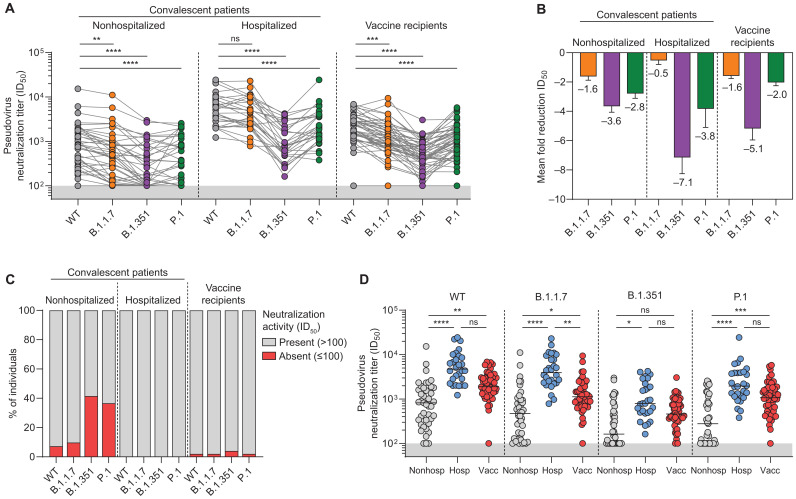
Neutralization of VOCs B.1.1.7, B.1.351, and P.1 by convalescent and vaccinee sera. (**A**) Half-maximal neutralization (ID_50_) titers of polyclonal sera from nonhospitalized convalescent patients (*n* = 41), hospitalized convalescent patients (*n* = 28), and vaccine recipients (*n* = 50) against pseudoviruses of WT and B.1.1.7, B.1.351, and P.1 VOCs. Connected dots indicate results from the same individual. The lower cutoff for neutralization was set at an ID_50_ of 100 (gray shading). (**B**) Means ± SEM fold reductions in ID_50_ titers for nonhospitalized convalescent patients, hospitalized convalescent patients, and vaccine recipients against B.1.1.7, B.1.351, and P.1 VOCs pseudoviruses in comparison to ID_50_ titers against the WT pseudovirus. (**C**) Percentage of individuals in each of the three groups in (B) that has no detectable serum neutralizing activity (ID_50_ < 100) against the indicated pseudoviruses. (**D**) ID_50_ titers of nonhospitalized patients, hospitalized patients, and vaccine recipients against WT pseudovirus and each of the VOC pseudoviruses. ****P < 0.0001, ***P < 0.001, **P < 0.01, and *P < 0.05. All data points shown here represent the mean of a technical triplicate and are representative of at least two independent experiments.

We next tested the neutralizing activity of convalescent and vaccinee sera (Fig. 2 and table S1). To this end, we generated lentiviral-based pseudoviruses of the currently widespread SARS-CoV-2 D614G (WT) variant as well as B.1.1.7, B.1.351 and P.1. We detected substantial neutralizing activity against the WT virus (half-maximal neutralization titer, ID_50_ > 100) in 96% of convalescent patients irrespective of hospitalization and in all vaccine recipients, except the one poor responder (Fig. 2A). The overall binding titers correlated well with the neutralization titers for all VOCs (fig. S1C). Nonhospitalized patients had the most heterogeneous responses, with WT neutralizing titers differing by up to ~150-fold, while titers against WT were much more homogeneous in hospitalized patients (up to ~20-fold difference) and vaccine recipients (up to ~12-fold difference) (Fig. 2A). Consistent with the binding results, we observed a marked and significant reduction in serum ability to neutralize VOC pseudoviruses (Fig. 2A). For all three groups, the difference was most apparent against the B.1.351 VOC, showing a reduction of ~4-, 7-, and 5-fold in neutralizing titers for nonhospitalized patients, hospitalized patients, and vaccine recipients, respectively (*P* < 0.0001; Fig. 2, A and B). The data were corroborated in an authentic SARS-CoV-2 neutralization assay (fig. S2, A to D). We did not observe an effect of medication, including the antiviral drug remdesivir and the anti-inflammatory drug dexamethasone, on neutralization activity in sera from convalescent but previously hospitalized patients (fig. S2E).

Some sera, most notably those from nonhospitalized patients, showed undetectable neutralization against some VOCs (defined as ID_50_ < 100), whereas they did neutralize WT pseudovirus (Fig. 2A). When the sera that reached the limit of detection against one of the VOCs (i.e., ID_50_ < 100) were excluded, the fold difference observed between WT and VOC neutralization remained very similar across the three groups (fig. S2F). Thirty-nine percent (16 of 41) nonhospitalized patients in this study lost all neutralizing activity against B.1.351, and 34% (14 of 41) were unable to neutralize P.1 at detectable levels, while they did neutralize WT pseudovirus and B.1.1.7 (Fig. 2C). In contrast, all hospitalized patients (28 of 28) retained at least some neutralizing activity against B.1.1.7, B.1.351, and P.1, and only 1 of 50 vaccine recipients lost neutralizing activity against B.1.351, suggesting that high titers of NAbs against WT are predictive for cross-neutralization of VOCs (Fig. 2C). Neutralizing titers of vaccine recipients against B.1.1.7 were an average ~ 3.6-fold lower than those of hospitalized patients (*P* = 0.0015), while this was less substantial and not significant for B.1.351 (~2.2-fold) and P.1 (~2.4-fold; Fig. 2D). Although B.1.351 showed the largest reduction in serum neutralization titers, the largest reduction in binding for both convalescent patients and vaccinee was seen against P.1 (Figs. 1C and 2B). Overall, we conclude that high neutralization levels against WT virus are predictive for the ability to neutralize VOCs, while low neutralization levels against WT often translate to the inability to neutralize VOCs B.1.351 and P.1 at detectable levels.

Although hospitalized patients did not show a significant reduction in B.1.1.7 neutralization, both vaccine recipients and nonhospitalized patients showed a small but statistically significant 1.6-fold reduction for B.1.1.7 compared to WT (*P* = 0.0035; Fig. 2, A and B). The sole RBD mutation in B.1.1.7, i.e., N501Y, might contribute to this effect, although it has only been sporadically implicated in NAb escape (*28*). Alternatively, the two NTD deletions in B.1.1.7 (Δ69-70 and Δ144; Fig. 1A) that are not present in WT, B.1.351, or P.1 might play a role. Moreover, while P.1 carries amino acid substitutions in the RBD at the exact same positions as B.1.351, the reduction in P.1 neutralization compared to WT was significantly less than for B.1.351 (*P* < 0.001; Fig. 2, A and B), which might be explained by differences in the NTD.

### Neutralizing monoclonal NAbs lose potency against VOCs

**Fig. 3.**
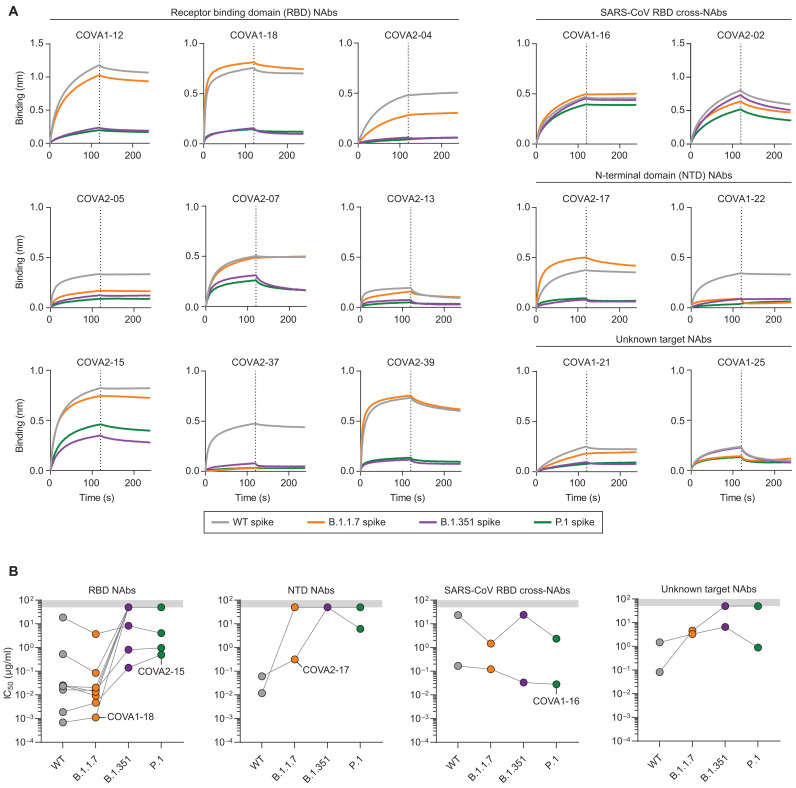
Binding kinetics and neutralization of NAbs isolated from convalescent patients with COVID-19. (**A**) BLI sensorgrams of 15 NAbs binding profile to WT spike and to each of the VOC S proteins (WT, gray; B.1.1.7, orange; B.1.351, purple; P.1, green). The dotted lines indicate the end of NAb association and the start of dissociation. (**B**) IC_50_ values of 15 NAbs to each of the pseudoviruses used in Fig. 2, separated by their target epitope on S protein. COVA1-18, COVA2-15, COVA2-17, and COVA1-16 are highlighted because of their neutralization characteristics. The gray shading indicates the maximum NAb concentration tested (50 μg/ml). Connected dots indicate IC_50_ values from the same NAb. Each dot represents the means ± SD of a technical triplicate. The curves shown are representative of at least two independent experiments.

To obtain more insight into the antibody specificities that were affected by the mutations in the VOCs, we assessed a panel of NAbs with known specificities isolated from convalescent patients, some of which have since been characterized structurally, and some of which have been evaluated in preclinical protection models (*25*, *29*, *30*). Biolayer interferometry (BLI) experiments showed that for most of RBD-targeting NAbs that span the four known epitope clusters on the RBD (*31*), the binding to B.1.351 and P.1 S protein was reduced substantially, while binding to the B.1.1.7 S protein was mostly similar to WT binding. This is consistent with observations that the only RBD mutation in B.1.1.7, i.e., N501Y, has not been associated with escape from antibodies and was probably selected for increased ACE2 binding (*8*, *9*), while E484K and K417N/T have a larger impact in neutralization by NAbs. RBD-targeting NAb COVA1-18, which neutralized WT with a half-maximal inhibitory concentration (IC_50_) in the nanogram-per-milliliter range and protected from WT infections in three preclinical animal models (*25*, *30*), displayed markedly reduced binding to B.1.351 and P.1 S proteins (Fig. 3A). Similarly, COVA2-15, which is highly potent against WT, showed decreased binding kinetics to S proteins from B.1.351 and P.1 compared to WT and B.1.1.7 S proteins (Fig. 3A). We also tested the binding of RBD-targeting, SARS-CoV cross-NAbs COVA1-16 and COVA2-02, which showed highly similar binding to all VOC S proteins tested, indicating that these NAbs particularly target a conserved epitope that has not been influenced by the mutations present in these three VOCs. This is in line with previous findings of COVA1-16 able to recognize RBDs of pangolin and bat origin, indicating that COVA1-16 recognizes an epitope that is highly conserved among sarbecoviruses (*29*). For the NTD-targeting NAbs COVA1-22 and COVA 2-17, we found a substantial reduction of binding to B.1.351 and P.1. COVA1-22 also showed reduced binding to B.1.1.7 S protein, while COVA2-17 retained binding to B.1.1.7. These results show that the binding of RBD NAbs and NTD NAbs is affected by mutations in VOCs, while there are some RBD NAbs that retain similar binding kinetics to all VOCs.

Next, we attempted to pinpoint which mutations have the most impact on binding of RBD NAbs. Therefore, we expressed soluble RBDs of VOCs B.1.1.7, B.1.351, and P.1 as well as a RBD with only the E484K mutation and performed an enzyme-linked immunosorbent assay (ELISA) with our panel of RBD NAbs (fig. S3, A and B). Similar to our BLI data (Fig. 3A), most RBD antibodies show a large and similar reduction in binding to B.1.351 and P.1 RBDs. However, COVA2-15 shows a ~4-fold lower half-maximal binding titer (EC_50_) to P.1 compared to B.1.351, indicating that the K417N mutation affects binding of this particular highly potent antibody more than K417T (fig. S3, A and B) (*25*). A similar observation can be made for COVA2-07, which does retain some binding against P.1 RBD but not against B.1.351 RBD (fig. S3, A and B). While E484K alone knocks out binding in COVA1-12, COVA1-18, COVA2-29, and COVA2-39, this is not the case for the other RBD NAbs that are not affected by E484K unless it is accompanied by N501Y and a substitution at position 417 (fig. S3, A and B). For SARS-CoV cross-NAbs COVA1-16, COVA2-02, and CR3022, none of the substitutions found in the VOCs studied here had any effect on binding. Thus, we show that while E484K alone has a substantial effect on the binding of some NAbs, it is the combination of E484K with other mutations such as K417N/T that have the greatest impact on the binding of RBD NAbs.

Last, we sought to confirm these findings in pseudovirus neutralization assays (Fig. 3B and fig. S4). In line with previous results (*25*), COVA1-18 and COVA2-15 exhibit picomolar IC_50_ titers against WT and B.1.1.7, while neutralization against B.1.351 was completely knocked out for COVA1-18 (i.e., IC_50_ > 50 μg/ml) and reduced by ~100-fold for COVA2-15 (Fig. 3B and fig. S4). Notably, only 5 of 11 tested RBD NAbs retained any detectable neutralization against B.1.351 and P.1, consistent with their abilities to bind the S proteins of these VOCs or not. Both NTD NAbs lost neutralization potency against B.1.351, while COVA2-17 retained neutralizing activity against P.1 (Fig. 3B). The fivefold decrease in neutralization potency for COVA2-17 against B.1.1.7 is consistent with earlier findings (*32*). Notably, SARS-CoV cross-NAb COVA1-16 did not lose potency against any VOCs, in line with previous results (*10*). The preservation of COVA1-16 potency (0.02 to 0.16 μg/ml for all VOCs) and its remarkable breadth are consistent with the epitope of COVA1-16 remaining virtually unchanged throughout sarbecovirus evolution and after >1 year of global SARS-CoV-2 evolution (*29*), thereby underlining the importance of having such NAbs readily available as therapeutics against emerging SARS-CoV-2 VOCs.

## DISCUSSION

In this study, we show that COVID-19 convalescent patients and mRNA vaccine recipients sampled at the expected peak of their immunity showed a marked decrease in binding and neutralization potency against two of three VOCs currently circulating (Fig. 2, A and B). While patients who experienced mild infection have lower binding and neutralization titers against the original virus and often no activity against B.1.351 and P.1, all hospitalized convalescent patients and all but one vaccine recipient maintain neutralizing titers against the three VOCs (Fig. 2C). Although the observed cross-reactivity is considerable, the substantial reduction in binding and neutralization titers against VOCs B.1.351 and P.1 could possibly leave them more vulnerable to a (re-)infection with these VOCs. First reports of breakthrough infection in vaccine recipients are starting to emerge (*33*).

To date, most studies have focused on a specific VOC or on a specific set of samples (i.e., vaccine recipients, convalescent patients, or NAbs) (*4*, *10*, *16*, *20*). Here, through systematic comparison of nonhospitalized patients, hospitalized patients, and vaccine recipients in the context of multiple VOCs, we are able to assess the impact of specific sets of mutations. We observed that serum binding titers were affected most by the P.1 VOC, while neutralizing titers against B.1.351 showed the largest reduction in all three groups studied (Figs. 1C and 2B). This irregularity might be explained by non-NAbs targeting the S2 domain or NTD being more affected by the amino acid substitutions in P.1 compared to B.1.351. The most notable differences between B.1.1.7 and the other two VOCs are the high number of mutations in the S2 domain and the lack of RBD mutations in B.1.1.7 that would result in a reduction in potency of RBD-targeting NAbs. Moreover, B.1.351 and P.1 show different neutralization signatures, most notably in hospitalized patients and vaccine recipients that have higher neutralization titers in general (Fig. 2B). While we and others (*34*) have shown that the E484K mutation affects binding and neutralization by serum and RBD NAbs greatly (fig. S3B), the discrepancy in neutralizing titers between B.1.351 and P.1 in vaccinees specifically might be because of the difference in amino acid substitution at position 417, as indicated by the NAb binding to the RBD variants. Alternatively, this discrepancy in neutralizing titers, despite similarity in their RBD, could be indicative of the NTD substitutions in B.1.351 causing a greater decrease in neutralizing titers than those found in P.1, which is reflected by NTD-targeting NAb COVA2-17. These results provide additional guidance for the risk evaluation of newly emerging variants during routine surveillance.

An implication of this study could be that (re-)infections with new VOCs will become more prevalent, especially later on when antibody titers have declined. However, the question remains whether infection with an emerging, antigenically altered variant will result in a less severe course of infection. While circulating NAbs are expected to play a dominant role in preventing infection, memory B cells might prevent severe disease, as they quickly gear up to generate new NAbs. Our finding that NAbs that have lost their neutralization capacity against VOCs can often still bind to VOC S proteins suggests that memory B cells of this specificity would still be able to recognize incoming VOCs and affinity-mature to regain neutralization capacity, resulting in faster virus clearance than during de novo immune responses. The first results of the Ad26.CoV2.S phase 3 clinical trial indicate that the vaccine, coding for the WT S protein, does result in a milder disease course after infection with the B.1.351 VOC (*35*). It remains to be seen if this effect persists when antibody levels decline over time.

Some real-world consequences from the observations we made here can be gleaned from phase 3 efficacy studies with vaccines based on the Wuhan Hu-1 strain and performed in geographic areas where VOCs dominated during the trial. First, the AstraZeneca ChAdOx1-S vaccine, which was tested in the United Kingdom when WT and B.1.1.7 were cocirculating, was subtly less effective against B.1.1.7 compared to the original virus (~70% versus ~81%) (*36*, *37*), while it was virtually ineffective in South Africa where B.1.351 was dominant during the trial (*38*). The Janssen COVID-19 vaccine was tested in many countries including the United States and South Africa. The vaccine was 74% effective at preventing moderate-to-severe disease in the United States, where 97% of sequenced viruses were of the reference Wuhan Hu-1 D614G strain, whereas in South Africa, 95% of sequenced viruses were B.1.351 and vaccine efficacy fell to 52% (*35*). Similar trends were reported for the Novavax subunit vaccine in a press release.

Our results and those of others (*35*, *36*, *38*) strongly suggest that booster vaccines with antigenically diverse SARS-CoV-2 variants are needed to induce long-lasting and more cross-reactive immunity. Multiple vaccine manufacturers have announced that they are already working on implementing VOCs into their immunization regimen, with Moderna having started the evaluation of a booster vaccine candidate based on B.1.351 in a phase 1 clinical trial (*39*). Considering the timelines for modification of mRNA versus other vaccine platforms, the mRNA vaccines might offer advantages against the evolving virus.

We observed that NAb COVA1-16 retained activity against B.1.351 and P.1, both of which have the E484K mutation that has now also been observed in the B.1.1.7 lineage and multiple other lineages (*9*, *12*). The intrinsic capacity of COVA1-16 to cross-neutralize SARS-CoV and its highly conserved epitope that remained unchanged throughout sarbecovirus evolution holds promise when considering SARS-CoV-2 evolution now and in the future. Moreover, COVA1-16 synergizes with another cross-reactive NAb CV38-142 to enhance neutralization of both SARS-CoV-2 and SARS-CoV (*40*). Together with the reduced effectiveness of several emergency use-authorized NAbs and NAb cocktails against B.1.351, our results re-emphasize the importance of having not only potent but also cross-reactive NAbs available as a therapeutic tool (*10*). Therefore, particularly when the potency of COVA1-16 can be improved, it could be an attractive component of a pan-sarbecovirus NAb cocktail to prevent and treat infection with emerging SARS-CoV-2 variants and to have on the shelf for future sarbecovirus pandemics.

While our study aims to provide an overview of many different aspects of pre-existing immunity against VOCs, there are some limitations. In this study, we present a vaccinee population that consists of primary HCWs that are generally of middle age, with only four (8%) participants being older than 60 (Table 1). However, we found no correlation between age and titers in convalescent patients against any of the VOCs tested. Moreover, we have focused on the neutralization potential of immune sera and did not examine antibody effector functions that have been implicated previously in the control of SARS-CoV-2 infection (*41*). Similar to other studies, our convalescent and vaccinee sera samples were collected 4 to 6 weeks after infection and 4 weeks after vaccination (i.e., at the peak of immunity), respectively. While we present findings that may have implications for additional booster vaccines and the monitoring of VOCs, we did not examine the aspect of waning antibody titers and how those might influence VOC binding and neutralization. Last, we have merely examined an early line of defense against infection with SARS-CoV-2 (i.e., serum antibody levels), while we expect memory B cells to play an important part in reinfections with SARS-CoV-2 VOCs. Swift reactivation of memory compartments may lead to reduced transmissibility, a milder course of disease and a more potent immune response.

In conclusion, we observed a substantial reduction in binding and neutralization potency against all three VOCs in most of the samples, with the individuals with lowest binding and neutralization titers losing potency together (i.e., ID_50_ < 100), especially to B.1.351 and P.1. These data have implications for the degree to which pre-existing immunity can protect against subsequent infection with VOCs, the possible need of vaccine modifications to increase immune coverage, and the use of monoclonal antibodies of therapeutics against SARS-CoV-2. An important outstanding question is what else the virus has in store for us and whether VOCs, when not brought under control, will evolve further and continue to escape from humoral immunity induced by infection or vaccination.

## MATERIALS AND METHODS

### Study design

The sera of 69 SARS-CoV-2–infected adults were collected 4 to 6 weeks after symptom onset through the cross-sectional COSCA cohort (NL 73281.018.20) as described previously (*25*). All participants had at least one nasopharyngeal or oropharyngeal swab positive for SARS-CoV-2 as determined by quantitative reverse transcriptase polymerase chain reaction (qRT-PCR; Roche LightCycler 480, targeting the envelope gene 113 base pairs). Participants were included from the start of the COVID-19 pandemic in the Netherlands in March 2020 until the end of January 2021. To investigate the humoral response to the Pfizer-BioNTech COVID-19 mRNA vaccine, we used sera collected 4 weeks after the second dose of 50 HCWs who were included at the beginning of the pandemic in March 2020 in a prospective serologic surveillance cohort in two tertiary medical centers in the Netherlands (S3 cohort; NL 73478.029.20). Participants of the surveillance cohort were tested routinely for seroconversion by using a WANTAI (WS-1096) SARS-CoV-2 RBD total immunoglobulin serum ELISA and underwent additional PCR testing when experiencing COVID-19–related symptoms. Participants were excluded when any of the above tests were indicative of a SARS-CoV-2 infection. Both studies were conducted at the Amsterdam University Medical Centers in the Netherlands and approved by the local ethical committee. All individuals included in this study gave written informed consent before participating.

### Protein design

The S constructs contained the following mutations compared to the WT variant (Wuhan Hu-1; GenBank: MN908947.3): deletion (Δ) of H69, V70 and Y144, N501Y, A570D, D614G, P681H, T716I, S982A, and D1118H in B.1.1.7; L18F, D80A, D215G, L242H, R246I, K417N, E484K, N501Y, D614G, and A701V in B.1.351; and L18F, T20N, P26S, D138Y, R190S, K417T, E484K, N501Y, D614G, H655Y, and T1027I in P.1. They were ordered as gBlock gene fragments (Integrated DNA Technologies) and cloned Pst I/Not I in a pPPI4 expression vector containing a hexahistidine (his) tag with Gibson Assembly (Thermo Fisher Scientific). All S constructs were verified by Sanger sequencing and subsequently produced in human embryonic kidney (HEK) 293F cells (Thermo Fisher Scientific) and purified as previously described (*25*). The RBD constructs contained the following mutations: N501Y in B.1.1.7; K417N, E484K, and N501Y in B.1.351; K417T, E484K, and N501Y in P.1; and E484K as a single mutant. They were made by introducing the mutations into the SARS-CoV-2 RBD (331 to 528 amino acids)–StrepII construct provided by D. Lakshamanane (*42*) using the QuikChange Site-Directed Mutagenesis Kit (Agilent Technologies). All RBD constructs were produced in ExpiCHO cells and purified as previously described (*43*).

### Multiplex protein microarray

Human CoV protein microarray (HCoV-PMA) slides were produced as described previously (*23*). WT and VOC (B.1.1.7, B.1.351, and P.1) S proteins were spotted in duplicate in three drops of 333 pl each on 24-pad nitrocellulose-coated slides (ONCYTE AVID, Grace Bio-Labs, Bend, USA) by using a noncontact marathon Arrayjet microarray spotter (Roslin, UK). Printed microarray slides were pretreated with BLOTTO blocking buffer (Thermo Fisher Scientific) to avoid nonspecific binding. Sera were tested in four fourfold dilutions starting at 1:20 and diluted in BLOTTO buffer containing 0.1% Surfact-Amps 20 (Thermo Fisher Scientific) as previously described (*23*). Subsequently, slides were incubated with goat anti-human immunoglobulin G (IgG), F(ab′)2 fragment specific, and Alexa Fluor 647 conjugated (Jackson Immunoresearch, West Grove, USA) and diluted 1:1000 in BLOTTO buffer with 0.1% Surfact-Amps 20 as described. Incubation steps were followed by a washing step with 1× phosphate-buffered saline (PBS) with 0.1% Tween 20. After the last wash, slides were washed with sterile water and dried. Day-to-day variations were monitored by including a SARS-CoV-2–positive control serum in each test round. An in-house SARS-CoV-2 standard was included in each test batch to correct for test-to-test variation. If the titer of the positive control deviated more than twofold from the expected titer, the test batch was rejected and repeated.

### Pseudovirus design

The B.1.1.7, B.1.351, and P.1 pseudovirus constructs contained the same mutations as the S constructs. They were ordered as gBlock gene fragments (Integrated DNA Technologies) and cloned Sac I/Apa I in the pCR3 SARS-CoV-2–S_Δ19_ expression plasmid (GenBank: MT449663.1) (*44*) using Gibson Assembly (Thermo Fisher Scientific). The D614G pseudovirus construct was made using the QuikChange Site-Directed Mutagenesis Kit (Agilent Technologies). All constructs were verified by Sanger sequencing. Pseudovirus was produced by cotransfecting the pCR3 SARS-CoV-2–S_Δ19_ expression plasmid with the pHIV-1_NL43_ ΔEnv-NanoLuc reporter virus plasmid in HEK293T cells (American Type Culture Collection, CRL-11268) (*44*, *45*). Cell supernatant containing the pseudovirus was harvested 48 hours after transfection and stored at −80°C until further use.

### Pseudovirus neutralization assay

HEK293T/ACE2 cells provided by P. Bieniasz (*44*) were seeded at a density of 20,000 cells per well in a 96-well plate coated with poly-l-lysine (50 μg/ml) 1 day before the start of the neutralization assay. NAbs (1 to 50 μg/ml) or heat-inactivated sera samples (1:100 dilution) were serial diluted in fivefold resp. threefold steps in cell culture medium [Dulbecco’s modified Eagle’s medium (Gibco) supplemented with 10% fetal bovine serum, penicillin (100 U/ml), streptomycin (100 μg/ml), and GlutaMAX (Gibco)], mixed in a 1:1 ratio with pseudovirus, and incubated for 1 hour at 37°C. These mixtures were then added to the cells in a 1:1 ratio and incubated for 48 hours at 37°C, followed by a PBS wash, and lysis buffer was added. The luciferase activity in cell lysates was measured using the Nano-Glo Luciferase Assay System (Promega) and GloMax system (Turner BioSystems). Relative luminescence units were normalized to the positive control wells where cells were infected with pseudovirus in the absence of NAbs or sera. The inhibitory concentration (IC_50_) and neutralization titers (ID_50_) were determined as the NAb concentration and serum dilution at which infectivity was inhibited by 50%, respectively, using a nonlinear regression curve fit (GraphPad Prism software version 8.3) (*45*). Samples with ID_50_ titers of <100 were defined as having undetectable neutralization.

### Authentic virus neutralization assay

SARS-CoV-2 virus neutralization assays were performed as described previously (*46*, *47*). Duplicates of twofold serial dilutions (starting at 1:10) of heat-inactivated sera (30 m, 56°C) were incubated with 100 median tissue culture infectious dose of SARS-CoV-2 strains hCoV-19/Netherlands/ZuidHolland_10004/2020, D614G (WT) and hCoV-19/Netherlands/ NoordHolland_10159/2021 (B.1.351, EVAg, catalog no. 014 V-04058) at 35°C for 1 hour in 96-well plates. Vero E6 cells were added in a concentration of 20,000 cells per well and incubated for 72 hours at 35°C. The serum virus neutralization titer was defined as the reciprocal value of the sample dilution that showed a 50% protection of virus growth. Samples with titers of ≥20 were defined as SARS-CoV-2 seropositive.

### Monoclonal antibodies

The NAbs used in this study were isolated from participants in the COSCA study as described before. NAbs were produced in HEK293F (Thermo Fisher Scientific) cells as previously described (*25*).

### Biolayer interferometry

BLI experiments were performed on an Octet K2 (ForteBio). Nickel–nitrilotriacetic acid biosensors (ForteBio) were loaded with 20 μg/ml of each of the four different his-tagged S proteins (WT, B.1.1.7, B.1.351, and P.1) in running buffer (PBS, 0.02% Tween 20, and 0.1% bovine serum albumin) for 300 s. After the biosensors were washed in a well containing running buffer to remove the excess protein, the biosensors were dipped in a well containing NAb (30 μg/ml) in running buffer for 120 s to measure association. Next, the biosensors were moved to a well containing running buffer for 120 s to measure the dissociation of the S protein–NAb complexes.

### Enzyme-linked immunosorbent assay

Purified RBD-StrepII proteins (200 ng per well) were coated overnight onto Nunc MaxiSorp 96-well plates (Thermo Fisher Scientific) at 4°C. The plates were washed three times with PBS/0.05% Tween 20 before blocking with 5% milk/PBS for 1 hour at room temperature (RT). After washing as described above, COVA NAbs, serially diluted in 2% milk/PBS, were added and incubated for 1 hour at RT. Bound NAbs were detected using horseradish peroxidase–conjugated goat anti-human IgG (1:3000) in 2% milk/PBS for 1 hour at RT. After washing, the color reaction was developed using 3,3′,5,5′-tetramethylbenzidine substrate (Thermo Scientific Scientific). The reaction was stopped by adding 0.3 N sulfuric acid, and OD_450_ (optical density at 450 nm) was read using an Enspire instrument (PerkinElmer).

### Visualization and statistical analysis

Data visualization and statistical analyses were performed in GraphPad Prism software (version 8.3). The nonparametric Friedman test was performed to assess statistical differences for paired samples, while the nonparametric Kruskal-Wallis test was performed for unpaired samples. Significance is denoted as ****P < 0.0001, ***P < 0.001, **P < 0.01, and *P < 0.05; ns, not significant.

## Supplementary Materials

### Other Supplementary Material for this manuscript includes the following:

Table S1

Members of the collective authorship cohort

## Notes

View/request a protocol for this paper from *Bio-protocol*.
