# Supplementary material for: Emerging SARS-CoV-2 variants of concern evade humoral immune responses from infection and vaccination
Source: Sci Adv. 2021 Sep 3;7(36):eabj5365. doi: 10.1126/sciadv.abj5365 (PMC8442901; doi:10.1126/sciadv.abj5365)
Supplement: Supplementary file 1 — Figs. S1 to S4 Legend for table S1 [file sciadv.abj5365_sm.pdf]

Science Advances
AAAS

Supplementary Materials for

Emerging SARS-CoV-2 variants of concern evade humoral immune responses from infection and vaccination

Tom G. Caniels, Ilja Bontjer, Karlijn van der Straten, Meliawati Poniman, Judith A. Burger, Brent Appelman, Ayesha H.A. Lavell, Melissa Oomen, Gert-Jan Godeke, Coralie Valle, Ramona Mögling, Hugo D.G. van Willigen, Elke Wynberg, Michiel Schinkel, Lonneke A. van Vught, Denise Guerra, Jonne L. Snitselaar, Devidas N. Chaturbhuj, Isabel Cuella Martin; Amsterdam UMC COVID-19 S3/HCW study group, John P. Moore, Menno D. de Jong, Chantal Reusken, Jonne J. Sikkens, Marije K. Bomers, Godelieve J. de Bree, Marit J. van Gils*, Dirk Eggink*, Rogier W. Sanders*

*Corresponding author. Email: r.w.sanders@amsterdamumc.nl (R.W.S.); dirk.eggink@rivm.nl (D.E.); m.j.vangils@amsterdamumc.nl (M.J.v.G.)

Published 3 September 2021, *Sci. Adv.* **7**, eabj5365 (2021)
DOI: 10.1126/sciadv.abj5365

The PDF file includes:

Figs. S1 to S4
Legend for table S1

Other Supplementary Material for this manuscript includes the following:

Table S1
Members of the collective authorship cohort

Fig. S1

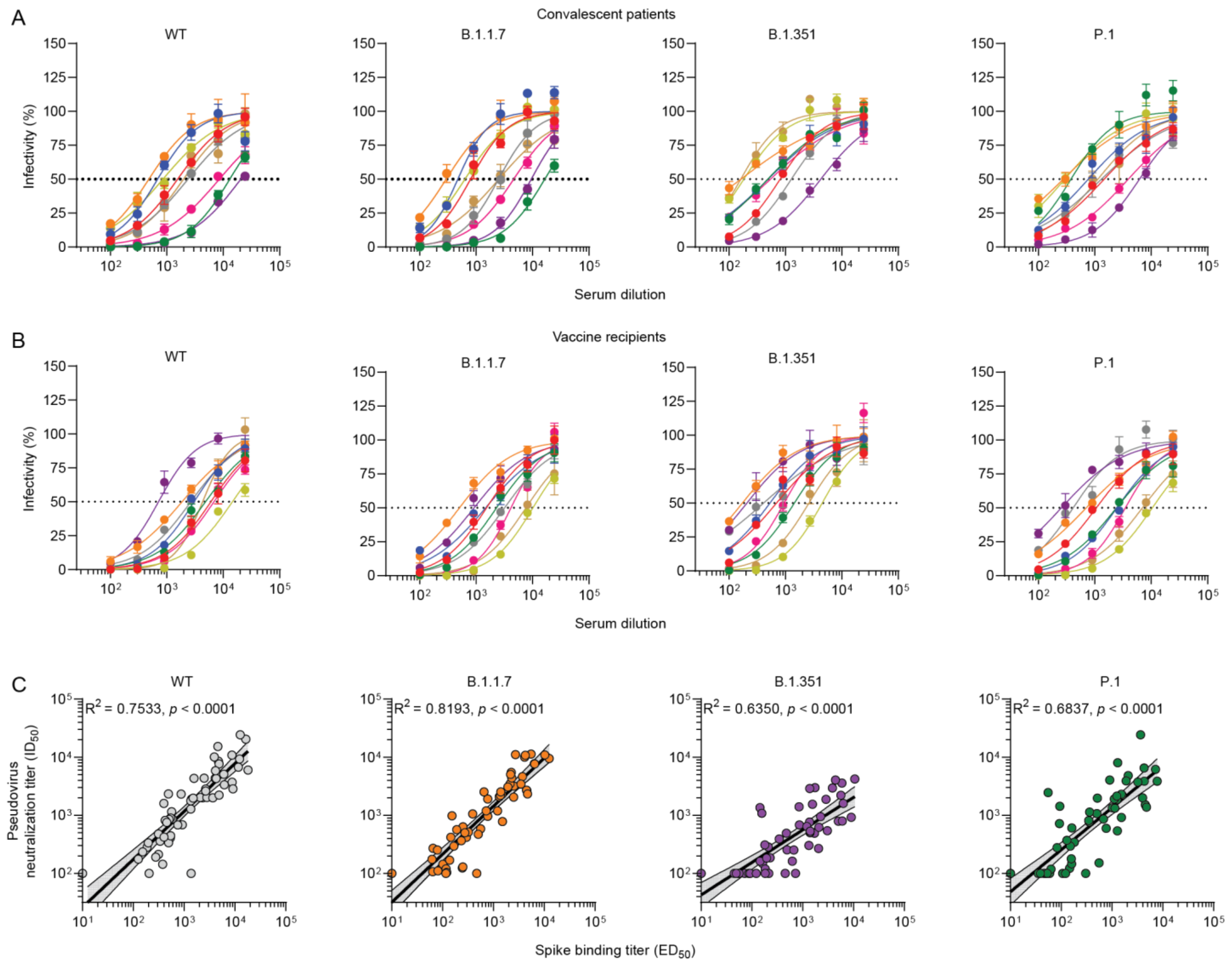

Supplementary Figure 1. Pseudovirus neutralization of convalescent individuals and vaccine recipients. Infectivity curves of WT, B.1.1.7, B.1.351 and P.1 SARS-CoV-2 pseudovirus tested with a subset of (**A**) convalescent sera and (**B**) vaccinee sera in 3-fold serial dilutions starting at 1:100 serum dilution. Each color indicates a different serum sample. Each dot represents the mean ± SD of a technical triplicate. (**C**) Correlation plots depicting the correlation between S protein binding (ED_{50}) titers and pseudovirus neutralization (ID_{50}) titers

for WT and B.1.1.7, B.1.351 and P.1 VOC. R^2 and P values are indicated on the graphs. The 95% confidence interval around the linear regression line (black) is shaded in grey.

Fig. S2

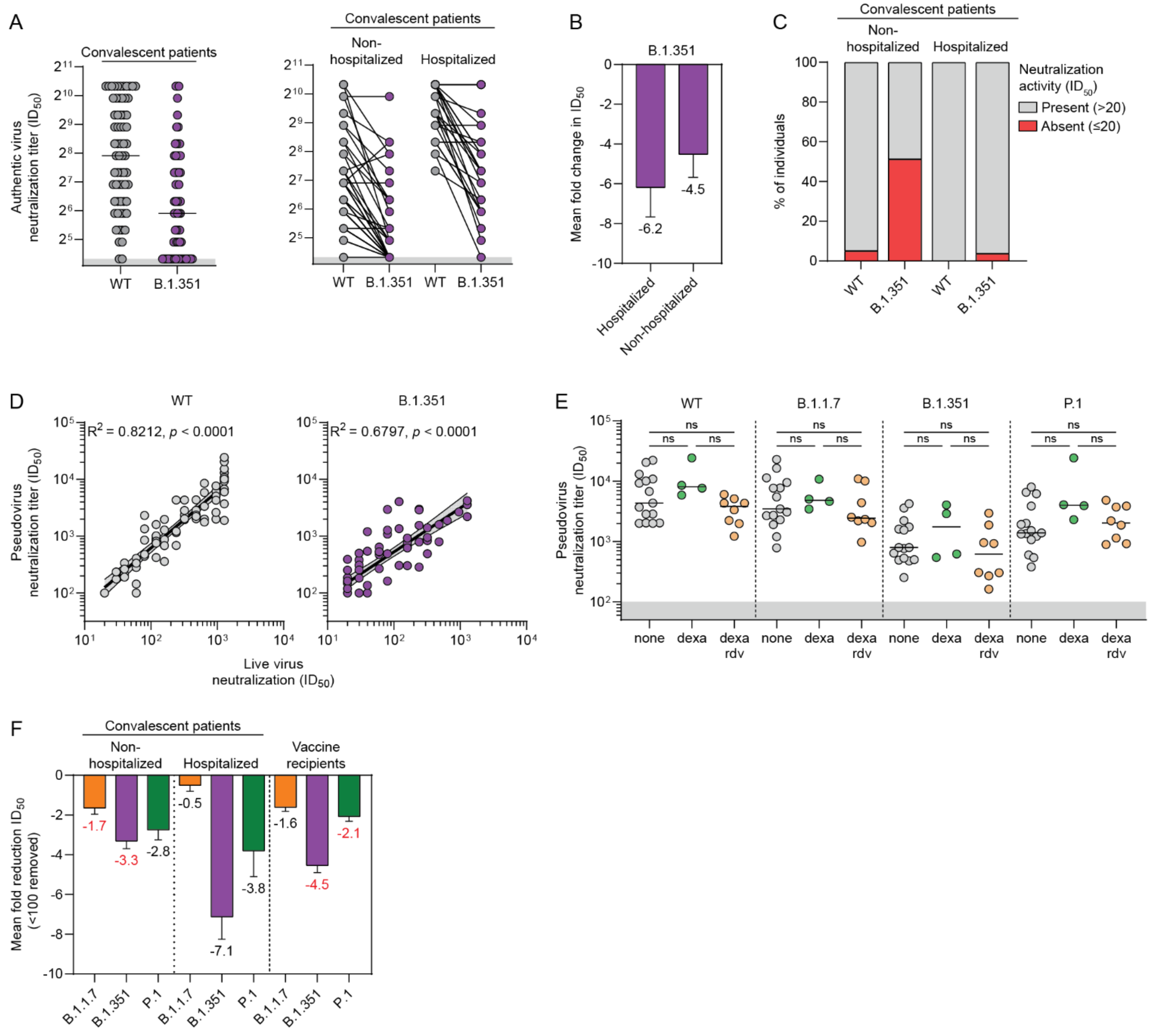

Supplementary Figure 2. Authentic virus neutralization and effect of medication on pseudovirus neutralization titers. (**A**) Median authentic virus neutralization (ID_{50}) titers for WT and B.1.351 for a subset of convalescent patients (left panel, n = 65) and for convalescent patients split by hospitalization status (right panel, n = 65). Grey shading indicates the lower limit of detection ($ID_{50} < 20$). (**B**) Mean ± SEM fold change in authentic virus neutralization titer

against B.1.351 in comparison to WT for hospitalized and non-hospitalized patients. (**C**) Percentage of individuals in each of the two groups in (**B**) that have no detectable serum neutralizing activity (ID_{50} < 20) against the indicated authentic viruses. (**D**) Correlation plots depicting the correlation between pseudovirus neutralization (ID_{50}) titers and authentic virus neutralization (ID_{50}) titers for WT and B.1.351. R^2 and P values are indicated on the graphs. The 95% confidence interval around the linear regression line (black) is shaded in grey. (**E**) Median pseudovirus neutralization titers against WT, B.1.1.7, B.1.351 and P.1 VOC in hospitalized patients grouped by received medication. Indicated on the x-axis is the medication they received. Dexa, dexamethasone; rdv, remdesivir. Grey shading indicates the limit of detection (ID_{50} < 100). (**F**) Mean ± SEM fold reductions in ID_{50} titers for non-hospitalized convalescent patients, hospitalized convalescent patients and vaccine recipients against B.1.1.7, B.1.351 and P.1 VOC pseudoviruses in comparison to ID_{50} titers against the WT pseudovirus excluding samples with titers below or at the limit of detection (ID_{50} < 100). Red font color indicates values that have been changed in comparison to Fig. 2B.

Fig. S3

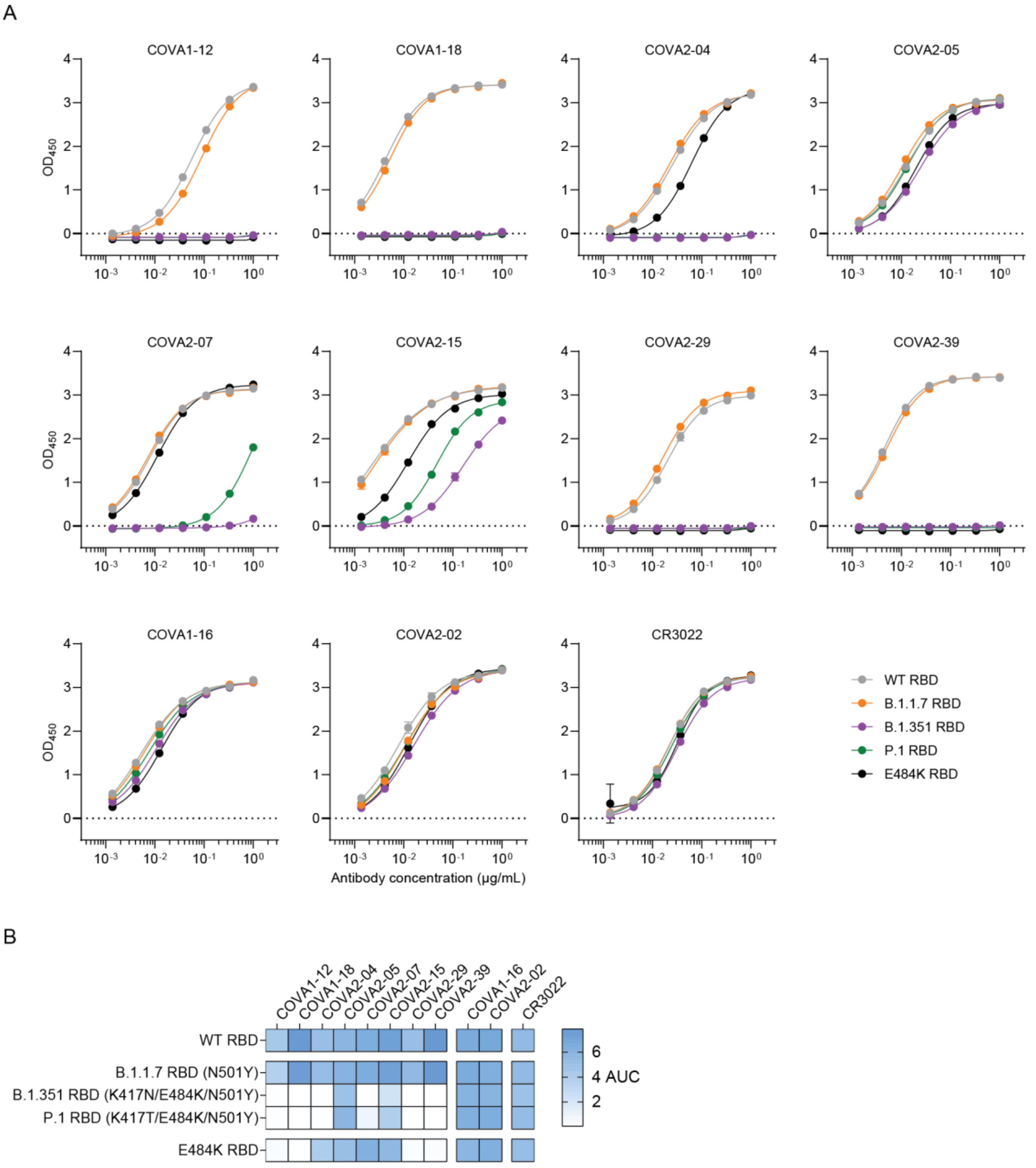

Supplementary Figure 3. Binding of NAbs to soluble receptor-binding domains (RBDs). (A) Enzyme-linked immunosorbent assay (ELISA) curves showing the binding of RBD targeting NAbs to WT, B.1.1.7, B.1.351, P.1 and E484K RBDs in duplicate. OD_{450}, optical density

measured at a wavelength of 450 nm. The dotted line indicates an OD_{450} of 0. (**B**) Area under the curve (AUC) values of the ELISA graphs in (**A**).

Fig. S4

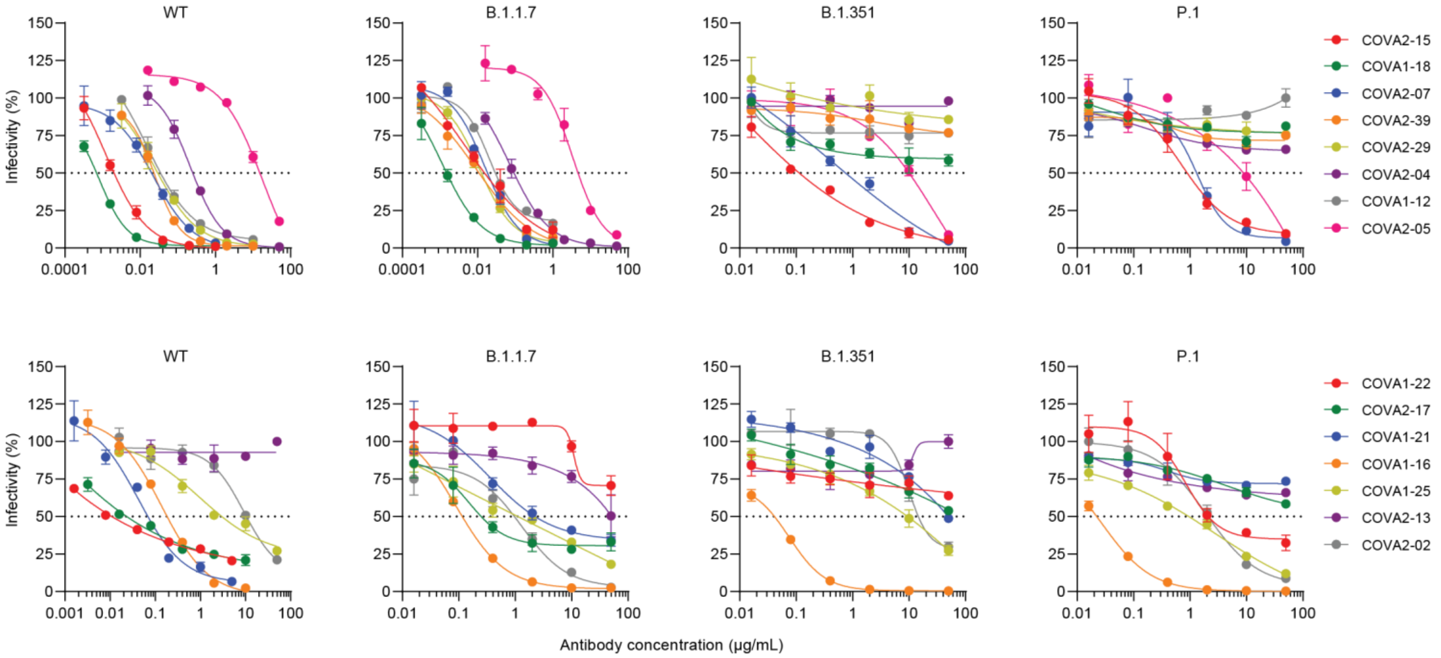

Supplementary Figure 4. Pseudovirus neutralization of a set of NAbs. Pseudovirus neutralization (ID_{50}) titers against WT, B.1.1.7, B.1.351 and P.1 for 15 NAbs. The top row depicts eight different NAbs, the bottom row depicts seven different NAbs. Each color in each graph indicates a different NAb. Each dot represents the mean ± SD of a technical triplicate.

Supplementary Table 1. Binding and neutralization titers for COVID-19 patients, vaccine recipients and NAbs against variants of concern.
