# Supplementary material for: Emerging SARS-CoV-2 variants of concern evade humoral immune responses from infection and vaccination
Source: Sci Adv. 2021 Sep 3;7(36):eabj5365. doi: 10.1126/sciadv.abj5365 (PMC8442901; doi:10.1126/sciadv.abj5365)
Supplement: Supplementary file 3 — Members of the collective authorship cohort [file sciadv.abj5365_members_of_the_collective_authorship_cohort.zip › sciadv.abj5365_Members_of_the_collective_authorship_cohort.docx]

**Members of the collective authorship cohort Amsterdam UMC COVID-19 S3/HCW study group**

Diederik van de Beek, Justin de Brabander, Matthijs C Brouwer, David TP Buis, Nora Chekrouni, Niels van Mourik, Sabine E Olie, Edgar JG Peters, Tom DY Reijnders, Alex R Schuurman, Marleen A Slim, Alexander PJ Vlaar, W Joost Wiersinga
